# Differential Immune-Modulating Activities of Cell Walls and Secreted Metabolites from Probiotic *Bacillus coagulans* JBI-YZ6.3 under Normal versus Inflamed Culture Conditions

**DOI:** 10.3390/microorganisms11102564

**Published:** 2023-10-15

**Authors:** Ifeanyi Iloba, Sage V. McGarry, Liu Yu, Dina Cruickshank, Gitte S. Jensen

**Affiliations:** 1 NIS Labs, 1437 Esplanade, Klamath Falls, OR 97601, USA; ifeanyi@nislabs.com; 2 NIS Labs, 807 St. George St., Port Dover, ON N0A 1N0, Canada; sage@nislabs.com (S.V.M.); yuliuliu1995@outlook.com (L.Y.); dina@nislabs.com (D.C.)

**Keywords:** anti-inflammatory, chemokines, cytokines, granulocyte colony-stimulating factor (G-CSF), monocytes, natural killer (NK) cells, T cells

## Abstract

Spore-forming probiotic bacteria, including *Bacillus coagulans*, are resilient and produce a variety of beneficial metabolites. We evaluated the immune-modulating effects of the novel probiotic strain *Bacillus coagulans* JBI-YZ6.3, where the germinated spores, metabolite fraction, and cell wall fraction were tested in parallel using human peripheral blood mononuclear cell cultures under both normal and lipopolysaccharide-induced inflamed culture conditions. The expression of CD25 and CD69 activation markers was evaluated via flow cytometry. Supernatants were tested for cytokines, interferons, chemokines, and growth factors using Luminex arrays. The germinated spores were highly immunogenic; both the cell wall and metabolite fractions contributed significantly. Under normal culture conditions, increased levels of immune activation were observed as increased expressions of CD25 and CD69 relative to natural killer cells, suggesting an increased ability to attack virus-infected target cells. On monocytes, a complex effect was observed, where the expression of CD25 increased under normal conditions but decreased under inflamed conditions. This, in combination with increased interleukin-10 (IL-10) and decreased monocyte chemoattractant protein-1 (MCP-1) production under inflamed conditions, points to anti-inflammatory effects. The production of the stem cell-related growth factor granulocyte colony-stimulating Factor (G-CSF) was enhanced. Further research is warranted to characterize the composition of the postbiotic metabolite fraction and document the characteristics of immunomodulating agents secreted by this probiotic strain.

## 1. Introduction

The gastrointestinal tract is home to a complex microbial community, and the mucosal lining and underlying tissue harbor a large proportion of the body’s innate immune cells. The cooperation of the innate immune system and the gut microbial community is required to maintain homeostasis [1]. Human physiology can be altered by the gut microbial community both with respect to health and disease. The disruption of the symbiotic relationship between the human body and its gut microbiome results in an unhealthy alteration of the composition of the microbiota, leading to a pro-inflammatory state that can adversely affect intestinal permeability, digestion, and metabolism, as well as immune responses [2,3].

Bacterial species from the spore-forming genus *Bacillus,* classified as Gram-positive facultative anaerobic bacteria, have gained increasing attention due to their novel probiotic potential [4]. There are 77 currently known *Bacillus* spp. that have shown positive health effects, including *B. coagulans, B. subtilis*, and *B. licheniformis* [5,6]. Among them, *B. coagulans* has gained recognition as a promising probiotic due to its status of being generally recognized as safe (GRAS) [7,8]. *B. coagulans* has a number of physiological characteristics that distinguish it from other probiotic *Bacillus* species, including growth conditions, biochemical reactions, the use of carbon sources, and a cell wall with high lipid contents compared to other Gram-positive bacteria [9,10]. Compared to non-spore-forming probiotic bacteria such as *Lactobacillus* spp., *B. coagulans* offers the advantage of improved stability during industrial processing and storage in functional foods [11,12]. The ability to withstand stomach acid and bile salts provides *B. coagulans* an edge for longer survival periods in the gastrointestinal tract [13,14,15,16]. The genomic analysis of *B. coagulans* shows no potential safety risks with regard to the transference of antibiotic resistance genes to other gut microbes [16,17,18]. In addition, *B. coagulans* has found wide application in the food industry because of its innate production of enzymes, vitamins, antimicrobials, amino acids, and short-chain fatty acids [19,20].

The immune-activating properties of probiotic cell walls are well documented, where the activation of host defenses includes the engagement of pattern recognition receptors (PPRs) on immune cells [21,22]. In addition, the metabolites secreted by *B. coagulans* have a variety of beneficial effects on the host (Table 1), including the increased production of mucin and tight junction proteins that protect the epithelial barrier [23,24,25,26], apoptosis-inducing substances that contribute to the destruction of cancer cells [27,28], antimicrobial peptides such as bacteriocin [29,30,31], and the fatty acid-mediated [32,33,34,35] and surfactants-mediated inhibition of pathogens [36,37], which prevent the formation of pathogenic biofilms. Furthermore, the metabolites stimulate the immune response by enhancing macrophage activity and modulating the secretion of immunoglobulins [38,39,40]. 

The secreted metabolites from probiotic bacteria are also described as postbiotics, which are defined as non-viable bacterial metabolic byproducts capable of conferring potential health benefits to the host. Different strains of *B. coagulans* are associated with different properties of secreted metabolites in terms of shifting the gut microbiome in favor of non-disease-related species. The production of bacteriocins by *Bacillus* species results in permeabilization and pore formation on target microbes, leading to the death of the target microbes. Bacteriocin secreted from *B. coagulans* had an inhibitory effect against *Escherichia coli* NCTC-10418, *Pseudomonas aeruginosa* NCIB-9016, *Klebsiella pneumoniae* NCIB-9111, *B. subtilis* NCTC-6346, *Staphylococcus aureus* NCTC7447, and fungi such as *Candida albicans* CBS-562 [41]. In addition, *B. coagulans* inhibited the growth of *Bacillus cereus* MTCC 430, *S. aureus* MTCC 3160, *Enterococcus* sp. MTCC 9728, *Lactobacillus* sp. MTCC 10093, and *Micrococcus luteus* MTCC 106, all of which are food-borne pathogens [42].

Previous research from our team involved another strain of *B. coagulans*, the *B. coagulans* GBI-30 6086 strain, and showed that the cell wall and metabolite fractions supported the maturation of antigen-presenting immune cells and modulated inflammatory processes in the gut by reducing proinflammatory cytokines [43,44]. Additionally, we showed that inactivated *B. coagulans* GBI-30 6086 cells activated human immune cells and altered the production of both immune activating and anti-inflammatory cytokines and chemokines, indicating that cell wall components remained viable and bioactive [45]. 

The present study investigated the effects of the germinated spores of the novel probiotic strain *B. coagulans* JBI-YZ6.3, in parallel with its metabolites and cell wall fractions, on immune activation and modulation under unstressed and inflamed conditions to determine whether the immunological consequences of exposure to the probiotic bacterium would differ depending on absence versus the presence of inflammation. This information is important for the development of effective probiotic formulations for specific health conditions in different populations.

**Table 1:** Properties of the cell walls and postbiotic metabolites from *Bacillus coagulans*.

Features	Function	Mechanisms of Action	References
Physical	Acidophilic	Intracellular pH is kept stable in acid environments by basic amino acids, proton-efflux systems, and highly impermeable cell membranes.	[13,14,15,16]
	Thermophilic	Thermal stability is attributed to the presence of saturated and straight-chain fatty acids, temperature-stable amino acids, and high guanine–cytosine content in DNA.	[14,15,16]
Cell wall	Immune modulation	Bacterial cell wall components bind to Toll-like receptors (TLRs) and NOD-like receptors (NLRs) and activate the host immune system.	[9,43,44,45]
**Metabolites**			
Bacteriocin	Anti-microbial	Target bacterial cell membranes are disrupted via the induction of cell permeabilization and pore formation.	[15,19,30]
Galactosidases	Improvement in carbohydrate metabolism.	Bacterial enzymes improve digestibility and carbohydrate metabolism by hydrolyzing non-digestible galactosides in food in the gut.	[18,20]
Fatty acids	Anti-fungal	Fungal cell membranes are disrupted using detergent-like properties of fatty acids and inhibiting the synthesis of membrane components such as ergosterol.	[9,32,33,35]
	Regulation of inflammation	Signaling molecules and chemical messengers are involved in gut-brain communication.	[46,47,48]
Biosurfactants	Anti-microbial	Pathogenic biofilm formation is inhibited on/in the gut mucosa.	[36,37]
Metabolites of unknown	Epithelial barrier protection	Stimulation of epithelial mucin secretion prevents microbial adhesion.	[23,24]
Chemical composition		Integrity of intestinal barriers is improved via the modulation of expression of tight junction proteins by host epithelial cells.	[23,24,25,26]
	Regulation of inflammation	Production of anti-inflammatory cytokine production by gut epithelial cells is stimulated.	[23,39,43,44,45]
	Anti-cancer effects	Cancer cell growth is inhibited via increased expression of pro-apoptotic genes.	[9,27,28]
	Antioxidant protection	Increased production of host antioxidant enzymes provides protection.	[39,49,50,51]

## 2. Materials and Methods

### 2.1. Reagents

Roswell Park Memorial Institute 1640 medium (Gibco cat. # 11835-030), penicillin–streptomycin 100× (Gibco cat. # 15140-122), fetal bovine serum (Gibco cat. # A38401-01), Dulbecco’s phosphate-buffered saline (PBS, Gibco cat. # 141190-136), lipopolysaccharide (LPS) (Invitrogen cat. # 00-4976-93), monoclonal antibodies CD3-SB702 (clone UCHT1, Invitrogen cat. #67-0038-42), CD56-phycoerythrin (clone CMSSB Invitrogen cat. # 12-0567-42), and CD69-fluorescein isothiocyanate (clone FN50, Invitrogen cat. #11-0699-42) were purchased from Thermo Fisher Scientific (Waltham, MA, USA). CD25-Brilliant Violet 421 (clone 2A3 BD cat. # 564033) and sodium heparin vacutainer tubes (BD cat. # 367878) were purchased from Becton-Dickinson (Franklin Lakes, NJ, USA). Bio-Plex Pro™ human cytokine arrays were purchased from Bio-Rad Laboratories Inc. (Hercules, CA, USA). Interleukin-2 (IL-2) (Sigma cat. # 17908-10KU) was purchased from Sigma-Aldrich Co. (St Louis, MO, USA). Lympholyte Poly (Cedarlane cat. # CL5070) was purchased from CedarLane (Burlington, NC, USA).

### 2.2. Bacillus Coagulans Germinated Spores, Metabolites, and Cell Wall Fractions

The test products, evaluated by addition to human immune cell cultures, were *Bacillus coagulans* germinated spores that were freshly prepared for each cell culture, and the metabolite and cell wall fractions were prepared in bulk and stored frozen in multiple aliquots so one aliquot could be thawed in preparation for each cell culture. The procedures are described below.

*Bacillus coagulans* spores were provided by the study sponsor, Jeneil Biotech, (Saukville, WI, USA). The strain is also available through the American Type Culture Collection, deposited as ATCC PTA-127366. The gene sequence of the bacterium is known and is available from GenBank (CP104390). The gene sequence of JBI-YZ6.3 was tested for antimicrobial resistance genes using two genome-wide screening programs, ResFinder and the Comprehensive Antibiotic Resistance Database (CARD), and screened for potentially toxigenic genes using known Bacillus toxin genes. No potential antibiotic resistance genes or toxin genes were detected in this strain.

Spores were prepared via aseptic fermentation with proprietary media and fermentation parameters to obtain a cell population comprised primarily of endospores, and they were verified via morphological observation with light microscopy. Cells were harvested from the fermentation media by centrifugation and dried under lyophilization in the absence of cryoprotectant. Enumeration was performed on dried biomass to measure spore counts (CFU/g). Dried biomass was standardized to a concentration of 1.5 × 10^10^ CFU/g by blending with identity-preserved maltodextrin.

**Figure 1:**
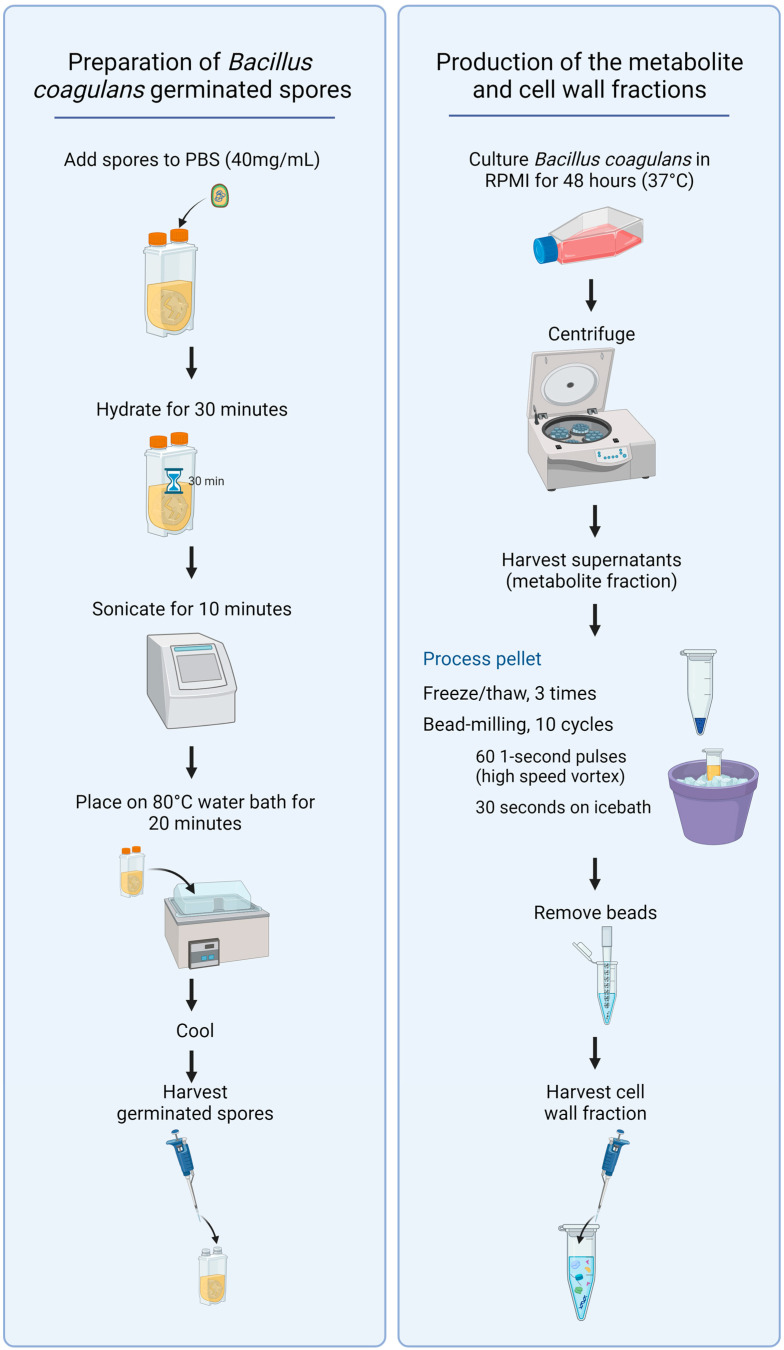
Diagram showing the procedures for preparing germinated spores, the metabolite fraction, and the cell wall fraction from *Bacillus coagulans*.

To produce germinated spores, a powder sample of dry spores containing 15 billion colony-forming units (CFU)/gram was weighed into sterile PBS (40 mg/mL) and shaken until a uniform suspension was generated (Figure 1, left panel). The suspension was incubated for 30 min at room temperature to allow the spores to hydrate. The suspension was sonicated for 10 min to reduce the number of aggregated spores. The sonicated suspension was transferred to a preheated water bath at 80 °C and incubated in the water bath for 20 min. The suspension was cooled immediately to 45 °C with intermittent vigorous shaking. This suspension was diluted 10-fold to create a stock solution and used to prepare serial dilutions for addition to cell cultures (1:10, 1:40, 1:160, and 1:640).

To produce samples of fermentation metabolites and cell wall fractions, a sample of germinated spores was cultured in Roswell Park Memorial Institute 1640 (RPMI-1640) culture medium under aerobic culture conditions at 37 °C for 48 h (Figure 1, right panel). The resulting culture was used to prepare both the metabolite fraction and the cell wall fraction for testing. 

The metabolite fraction was prepared from the 48 h culture via centrifugation. The supernatant containing the fermentation metabolites was decanted from the pellet and harvested. 

The pellet was used to prepare the cell wall fraction. The pelleted bacteria were washed three times in PBS, followed by 3 freeze–thaw cycles and subsequent bead-milling using low-protein binding 100-micron zirconium beads. The bead-milling consisted of 10 cycles, where each cycle involved 60 1 s pulse-vortexing to grind up the bacterial particles. Each 1 s of pulse-vortexing was followed by immediate immersion into an ice bath for 30 s to avoid unnecessary heating during the bead-milling cycles. The beads were allowed to settle, and the suspension of bacterial cell wall material was transferred to a clean tube, pelleted by centrifugation, the supernatant discarded, and the cell wall fraction resuspended in sterile PBS. 

For both the cell wall and metabolite fractions, multiple aliquots were prepared in PBS + penicillin (100 units/mL) and streptomycin (100 units/mL). The aliquots were frozen at −30 °C, and 1 aliquot was thawed on each lab testing day. After thawing, samples were vortexed and used for testing in immune cell cultures.

### 2.3. Immune Cell Activation

Peripheral venous blood was drawn from three healthy human donors upon written informed consent, as approved by the Sky Lakes Medical Center Institutional Review Board, Federalwide Assurance 2603. The blood was drawn into heparin vacutainer vials, and peripheral blood mononuclear cells (PBMCs) were isolated using Lympholyte Poly by centrifugation for 35 min at 400× *g*. PBMCs were washed twice in PBS and counted, and density was adjusted to establish cultures with a cell density of 10^6^/mL using Roswell Park Memorial Institute 1640 medium containing 10% heat-inactivated fetal calf serum and penicillin–streptomycin.

Serial dilutions of test products were added to PBMC cultures in U-bottom 96-well cell culture plates (NUNClon Delta Surface cat# 163320) at a density of 10^6^ cells/mL and a volume of 0.2 mL. Two parallel sets of cultures were prepared: (1) normal (un-stressed) culture conditions and (2) inflamed culture conditions, where the cells were treated with test products for 10 min, after which inflammatory conditions were triggered by the addition of 0.01 mL LPS per well for a final dose of 5 µg/mL LPS in cell cultures. Cultures were incubated at 37 °C and 5% CO_2_ for 16 h. 

LPS from *Escherichia coli* was used as a positive control for immune-cell activation. In parallel, interleukin-2 (IL-2) was used as a positive control for natural killer (NK) cell activation at a concentration of 100 IU/mL in cell culture. Untreated negative control cultures consisted of PBMCs exposed to phosphate-buffered saline in the absence of test products. All treatments, including each dose of the test product and each positive and negative control, were tested in triplicate. After 16 h, blood cells were isolated from each culture well and stained for 15 min with fluorochrome-labeled monoclonal antibodies at doses predetermined by titration. PBMC were then fixed in 0.5% formalin and acquired by flow cytometry using an Attune acoustic-focusing flow cytometer (Thermo Fisher Scientific, Waltham, MA, USA). Data analysis utilized gating on forward/side scatter to characterize lymphocyte and monocyte populations. In the lymphocyte population, 4 subsets were evaluated for expression of the CD25 and CD69 activation markers: CD3+ CD56− T cells, CD3+ CD56+ NKT cells, CD3− CD56+ NK cells, and the CD3-CD56− non-NK non-T cells.

### 2.4. Production of Cytokines, Chemokines, and Growth Factors

After 16 h of incubation, the supernatants were harvested from the PBMC cultures described above. The levels of 27 cytokines and chemokines were quantified using Bio-Plex protein arrays (Bio-Rad Laboratories Inc., Hercules, CA, USA) and utilizing xMAP technology (Luminex, Austin, TX, USA). The cytokine array included the following: IL-1β, IL-1ra, IL-2, IL-4, IL-5, IL-6, IL-7, IL-8, IL-9, IL-10, IL-12 (p70), IL-13, IL-15, IL-17, Eotaxin, basic FGF, G-CSF, GM-CSF, IFN-γ, IP-10, MCP-1 (MCAF), MIP-1α, MIP-1β, PDGF-BB, RANTES, TNF-α, and VEGF.

### 2.5. Statistical Analysis

The average and standard deviation for each data set were calculated using Microsoft Excel for Microsoft 365 MSO (Version 2309) (Microsoft Corporation, Redmond, WA, USA). Statistical analysis of in vitro data was performed using the 2-tailed, independent *t*-test. Statistical significance was set at *p* < 0.05, and a high level of significance was set at *p* < 0.01.

## 3. Results

### 3.1. Induction of the CD25 and CD69 Activation Markers on Immune Cell Subsets

**Figure 2:**
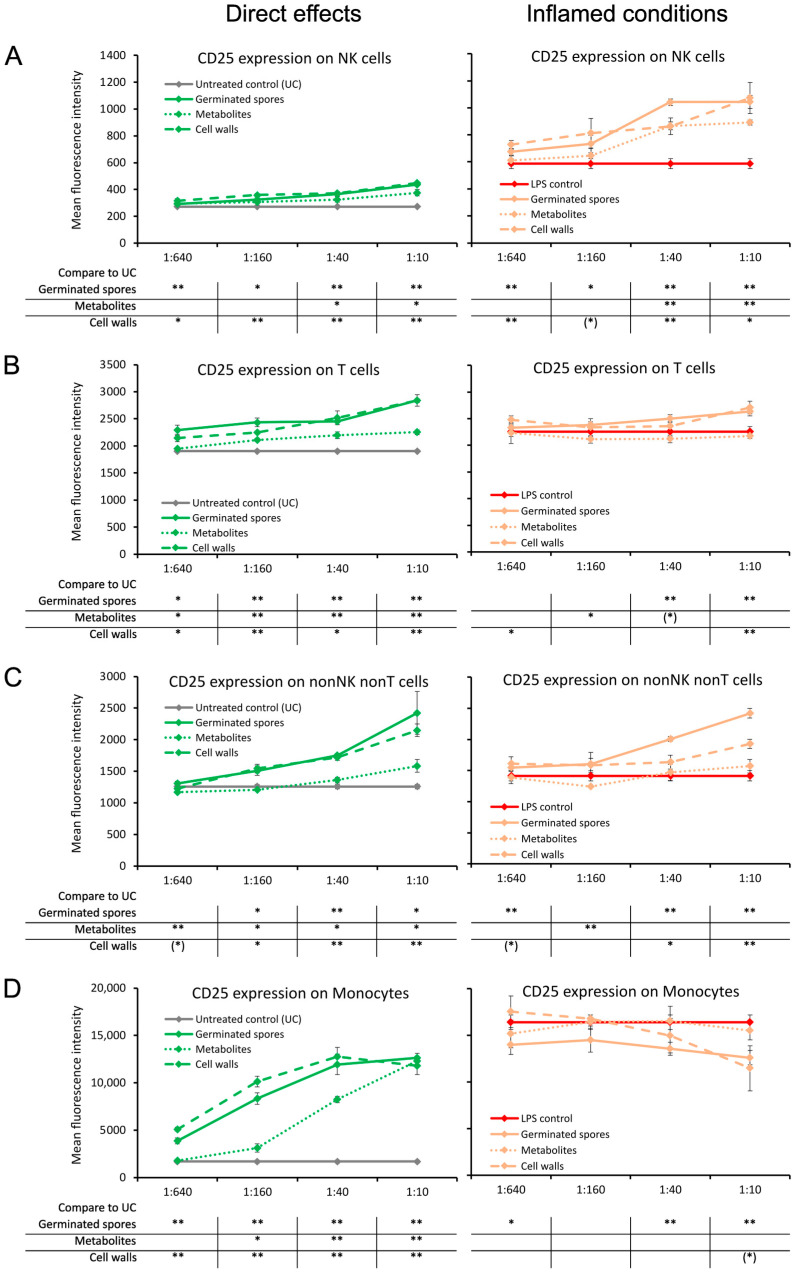
Expression levels of the CD25 activation marker on subsets of human peripheral blood mononuclear cells. The left panel (green lines) shows the direct effects of the germinated spores, metabolites, and cell walls; untreated control cultures (UCs) were used as negative controls (grey lines). The right panel (orange lines) shows the effects of the test products under inflamed culture conditions, where inflammation was induced by lipopolysaccharides (LPSs); LPS alone was used as a positive control (red lines). (**A**) Expression of CD25 on natural killer (NK) cells was mildly induced by all 3 products; under inflamed culture conditions, the products triggered an increase in LPS-induced levels of CD25. (**B**) Expression of CD25 on T cells was induced by all 3 products; under inflamed culture conditions, the germinated spores and the cell walls triggered a mild increase in LPS-induced levels of CD25, whereas the metabolites had no effect. (**C**) Expression of CD25 on nonNK nonT cells was induced by all 3 products; under inflamed culture conditions, the germinated spores triggered a robust increase, and the cell walls triggered a mild increase in the LPS-induced levels of CD25, whereas the effects of the metabolites were minimal. (**D**) Expression of CD25 on monocytes was induced by all 3 products; under inflamed culture conditions, the germinated spores triggered a robust decrease, and the cell walls triggered a mild decrease in the LPS-induced levels of CD25, whereas the metabolites had no effect. Statistical comparisons are shown in the tables below each graph. For the graphs on the left side, statistical comparisons are carried out on the untreated control cultures (UCx). For the graphs on the right side, statistical comparisons are carried out on the LPS-treated (LPS) control cultures. The statistical significance when comparing the test products to controls is indicated with (*) for *p* < 0.1, * for *p* < 0.05, and ** for *p* < 0.01.

The CD25 activation marker, also known as the alpha-chain of the heterotrimer IL-2 receptor, was induced by the germinated spores and the cell wall fraction under both normal and inflamed culture conditions (Figure 2). Compared to untreated control cultures, increased levels of CD25 expression were observed in natural killer (NK) cells; T cells; the non-NK non-T cell fraction, which contains dendritic cells; and monocytes. The increased levels of CD25 on all four cell subsets reached a high level of statistical significance for the cell wall fraction when compared to untreated control cultures (*p* < 0.01).

The increased levels of CD25 expression were milder in cultures treated with the metabolite fraction, for NK cells, T cells, and the non-NK non-T cell population (Figure 2A–C). In contrast, the two highest doses of the metabolite fraction induced highly significant increases in CD25 expression in the monocyte population compared to untreated control cultures (*p* < 0.01; Figure 2D).

Under inflamed conditions, CD25 expression was increased in NK cells by all three test products, reaching a high level of statistical significance at the second dose compared to the LPS control (*p* < 0.01; Figure 2A). CD25 expression levels were increased by both the germinated spores and cell wall fraction with respect to T cells and non-NK non-T cells, reaching a high level of statistical significance at the highest dose (*p* < 0.01; Figure 2B,C). The germinated spores and the cell wall fraction triggered the down-regulation of CD25 expression on monocytes under inflamed conditions (Figure 2D). In contrast, the metabolite fraction had no effect on CD25 expression levels on T cells, non-NK non-T cells, or monocytes under inflamed conditions (Figure 2B–D).

**Figure 3:**
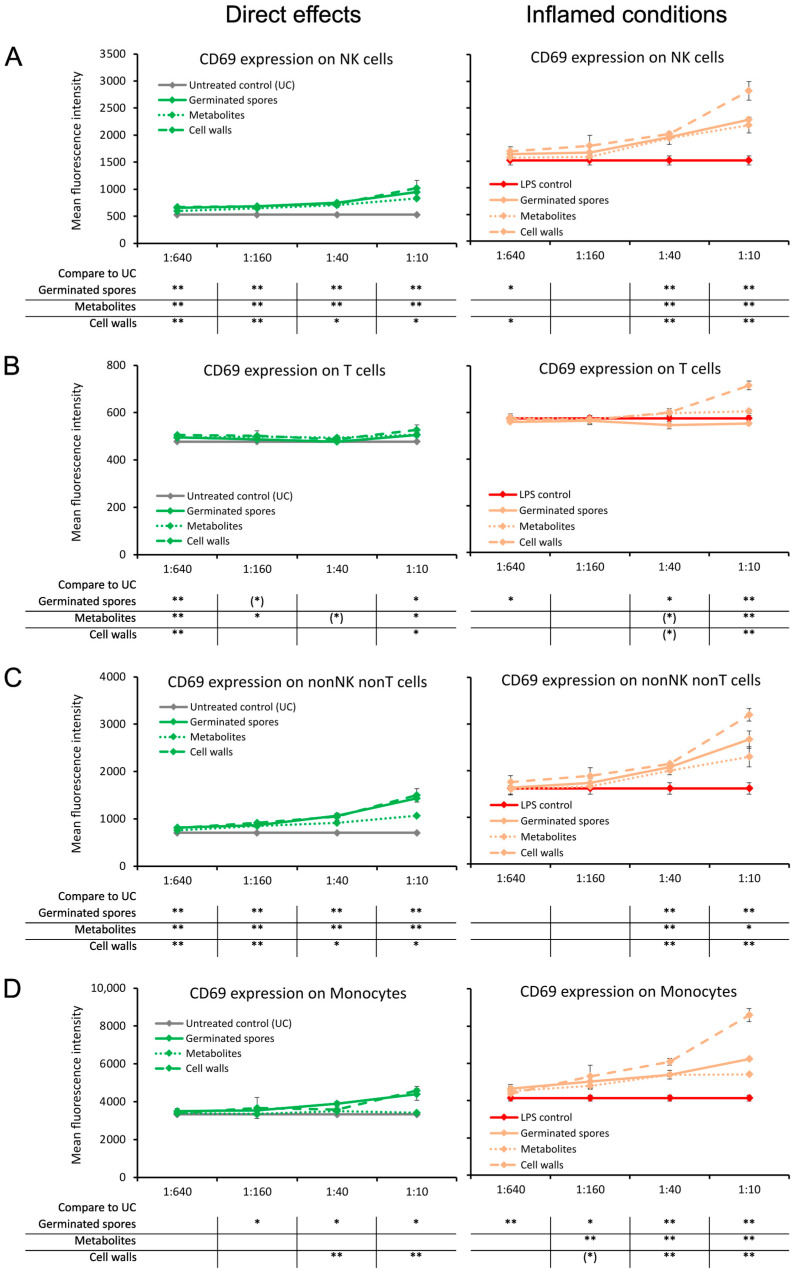
Expression levels of the CD69 activation marker on subsets of human peripheral blood mononuclear cells. The left panel (green lines) shows the direct effects of the germinated spores, metabolites, and cell walls; untreated control cultures (UCs) were used as negative controls (grey lines). The right panel (orange lines) shows the effects of the test products under inflamed culture conditions, where inflammation was induced by lipopolysaccharide (LPS); LPS alone was used as a positive control (red lines). (**A**) Expression of CD69 on natural killer (NK) cells was mildly induced by all 3 products; under inflamed culture conditions, the products triggered an increase in LPS-induced levels of CD69, and the effect was the strongest for the cell walls. (**B**) Expression of CD69 on T cells was not affected by the 3 products; under inflamed culture conditions, the highest dose of the cell walls triggered a mild increase in the LPS-induced levels of CD69. (**C**) Expression of CD69 on non-NK non-T cells was induced by all 3 products; under inflamed culture conditions, the cell walls triggered a robust increase, with milder effects from the germinated spores and metabolites. (**D**) Expression of CD69 on monocytes was induced by the germinated spores and the cell walls but not affected by the metabolites; under inflamed culture conditions, the cell walls triggered a robust increase, and the germinated spores and metabolites triggered a milder increase in LPS-induced levels of CD69. Statistical comparisons are shown in the tables below each graph. For the graphs on the left side, the statistical comparisons are for the untreated control cultures (UCs). For the graphs on the right side, the statistical comparisons are for the LPS-treated (LPS) control cultures. The statistical significance when comparing the test products to controls is indicated with (*) for *p* < 0.1, * for *p* < 0.05, and ** for *p* < 0.01.

The CD69 activation marker, a transmembrane C-Type lectin protein known to be upregulated by NF-κB signaling at the onset of an immune response, was induced on lymphocytes by all three test products under normal culture conditions (Figure 3). Compared to the untreated control cultures, CD69 expression was strongest on NK cells and non-NK non-T cells and almost absent on T cells (Figure 3A–C). The levels of CD69 expression on monocytes under normal conditions were increased by the germinated spores (*p* < 0.05) and the cell wall fraction (*p* < 0.01) but were not affected by the metabolite fraction when compared to untreated control cultures (Figure 3D).

Under inflamed conditions, CD69 expression was increased on all cell types by the cell wall fraction (*p* < 0.01; Figure 3A–D). The germinated spores and the metabolite fraction also triggered increased CD69 expressions under inflamed conditions on NK cells, non-NK non-T cells, and monocytes but had no effect on CD69 expression in T cells (Figure 3A–D).

### 3.2. Increased Pro-Activating Cytokine Production

**Figure 4:**
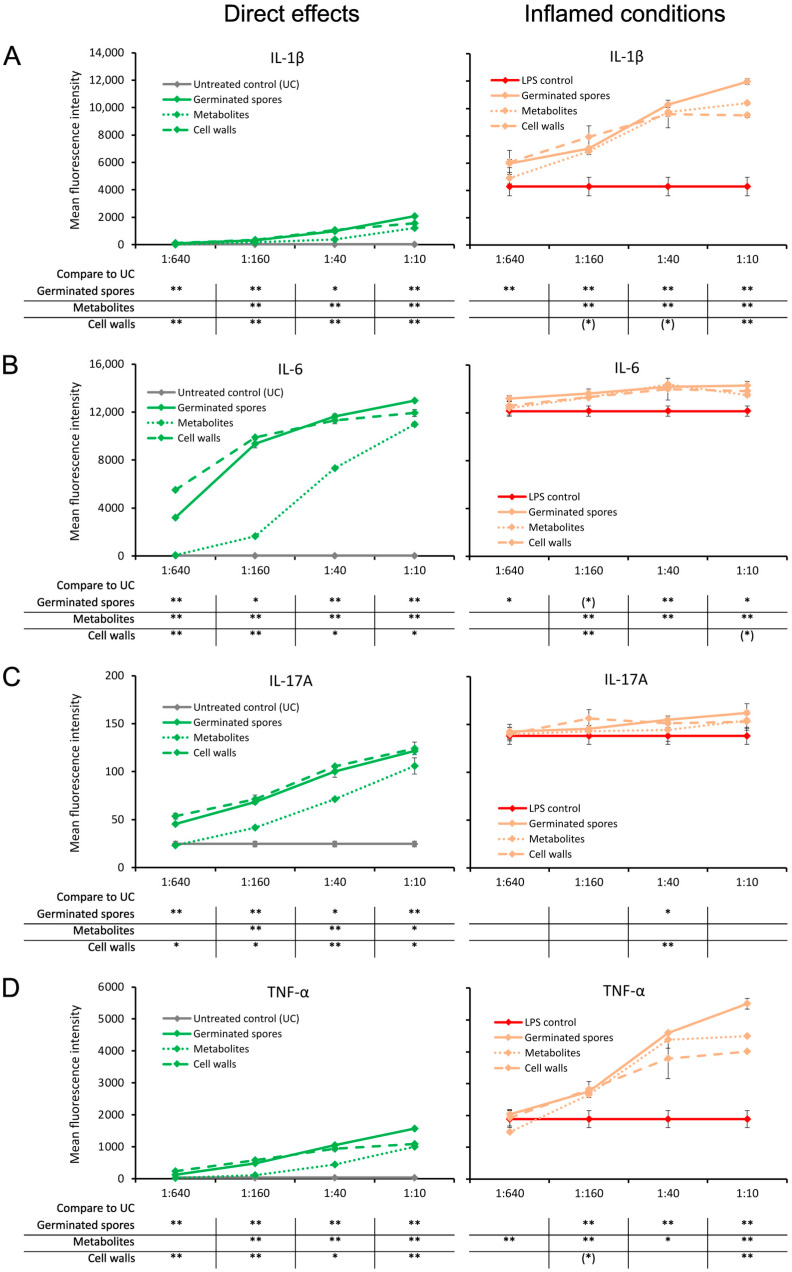
Cytokine production in cell cultures of human peripheral blood mononuclear cells. The left panel (green lines) shows the direct effects of the germinated spores, metabolites, and cell walls; untreated control cultures (UCs) were used as negative controls (grey lines). The right panel (orange lines) shows the effects of the test products under inflamed culture conditions, where inflammation was induced by lipopolysaccharides (LPSs); LPS alone was used as a positive control (red lines). (**A**) Interleukin-1-beta (IL-1β) was mildly induced by all 3 products, and under inflamed culture conditions, it triggered a robust increase in the LPS-induced levels of IL-1β. (**B**) Interleukin-6 (IL-6) was robustly induced by all 3 products, and under inflamed culture conditions, it triggered a mild but highly significant increase in the LPS-induced levels of IL-6. (**C**) Interleukin-17A (IL-17A) was robustly induced by all 3 products, and under inflamed culture conditions, it triggered a mild increase in the LPS-induced levels of IL-17A. (**D**) Tumor necrosis factor-alpha (TNF-α) was mildly induced by all 3 products, and under inflamed culture conditions, it triggered a robust increase in the LPS-induced levels of TNF-α. Statistical comparisons are shown in the tables below each graph. For the graphs on the left side, the statistical comparisons are for the untreated control cultures (UCs). For the graphs on the right side, the statistical comparisons are for the LPS-treated (LPS) control cultures. The statistical significance when comparing the test products to controls is indicated with (*) for *p* < 0.1, * for *p* < 0.05, and ** for *p* < 0.01.

The germinated spores and the cell wall fraction had similar effects under normal unstressed culture conditions, inducing a dose-dependent upregulation of the following immune-activating cytokines: interleukin-1β (IL-1β), interleukin-6 (IL-6), interleukin-17A (IL-17A), and tumor necrosis factor-alpha (TNF-α) (Figure 4). The metabolite fraction also induced the expression of these four cytokines, but the effect was milder (Figure 4).

When the test products were added to PBMC cultures in the context of an inflammatory challenge, the levels of these four cytokines were significantly higher than for the LPS control, but they were most robust for IL-1β and TNF-α. When comparing the TNF-α levels induced by the three test products, the germinated spores induced the highest level of TNF-α, and the cell wall fraction induced the lowest TNF-α levels compared to the LPS control (Figure 4D). The metabolite fraction induced higher levels of TNF-α under inflamed culture conditions than the cell wall fraction (Figure 4D).

### 3.3. Increased Interferon and Chemokine Production

**Figure 5:**
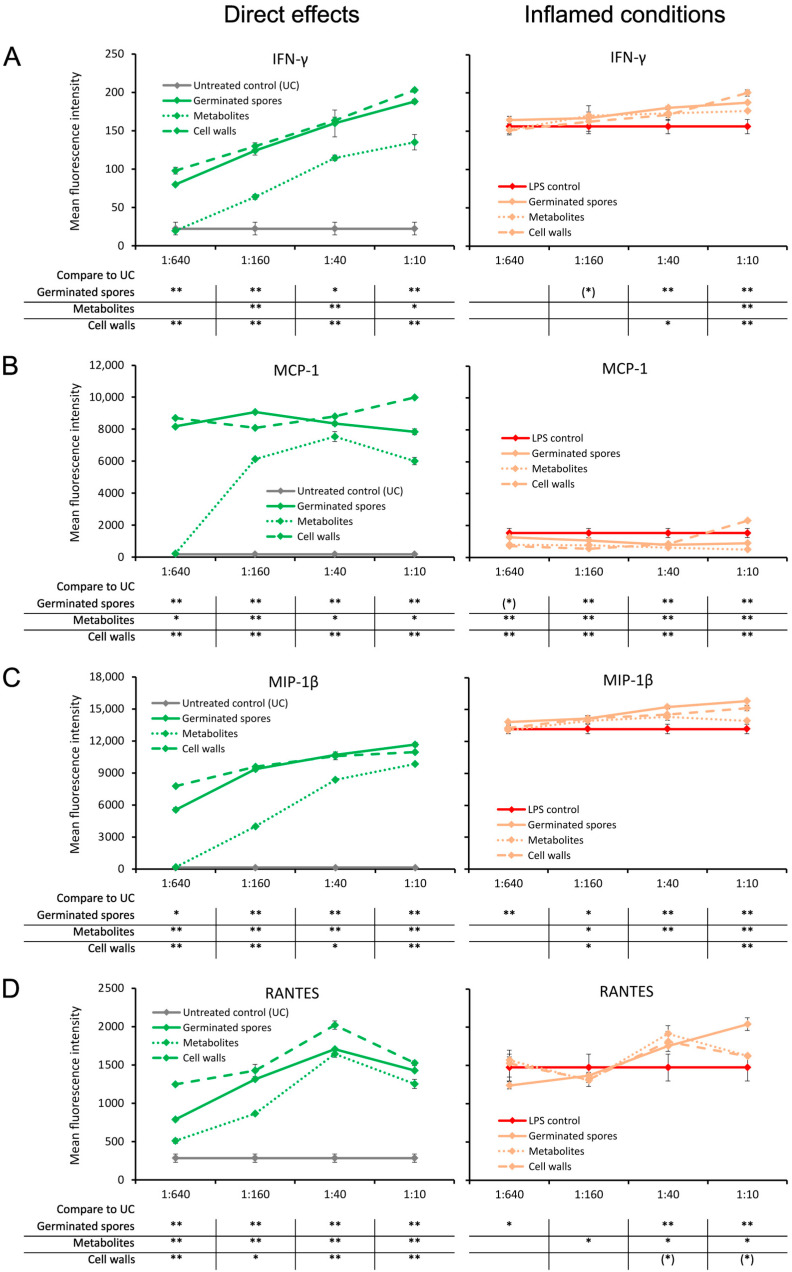
Cytokine production in cell cultures of human peripheral blood mononuclear cells. The left panel (green lines) shows the direct effects of the germinated spores, metabolites, and cell walls; untreated control cultures (UCs) were used as negative controls (grey lines). The right panel (orange lines) shows the effects of the test products under inflamed culture conditions, where inflammation was induced by lipopolysaccharides (LPSs); LPS alone was used as a positive control (red lines). (**A**) Interferon-gamma (IFN-γ) was robustly induced by all 3 products, and under inflamed culture conditions, they triggered a very mild increase in the LPS-induced levels of IFN-γ. (**B**) Monocyte chemoattractant protein-1 (MCP-1) was robustly induced by all 3 products; however, under inflamed culture conditions, the products triggered a decrease in the LPS-induced levels of MCP-1. (**C**) Macrophage inflammatory protein-1 beta (MIP-1β) was robustly induced by all 3 products, and under inflamed culture conditions, it triggered a mild increase in the LPS-induced levels of MIP-1β. (**D**) The regulated upon activation, normal T cell expressed and secreted (RANTES) chemokine was robustly induced by all 3 products; under inflamed culture conditions, the products triggered a mild increase in the LPS-induced levels of RANTES, which was the strongest for the germinated spores. Statistical comparisons are shown in the tables below each graph. For the graphs on the left side, the statistical comparisons are for the untreated control cultures (UCs). For the graphs on the right side, the statistical comparisons are for the LPS-treated (LPS) control cultures. The statistical significance when comparing the test products to controls is indicated with (*) for *p* < 0.1, * for *p* < 0.05, and ** for *p* < 0.01.

The germinated spores and the cell wall fraction had similar effects under normal unstressed culture conditions, inducing a dose-dependent upregulation of interferon-gamma (IFN-γ) and three chemokines: monocyte chemoattractant protein-1 (MCP-1), macrophage inflammatory protein-beta (MIP-1β), and regulated upon activation, normal T cell expressed and secreted (RANTES) (Figure 5). The metabolite fraction also induced these four cytokines, but the effect was milder (Figure 5). 

Under inflamed culture conditions, the levels of IFN-γ and MIP-1β were slightly higher in the cultures that were pre-treated with test products prior to inflammation. The mild increase was highly significant for the highest dose of all three test products compared to the LPS-treated control (*p* < 0.01; Figure 5A,C).

In addition, the test products increased the level of RANTES under inflamed culture conditions compared to the LPS-treated control cultures at the two higher doses (Figure 5D). This increase in RANTES production was highly significant for the germinated spores (*p* < 0.01) and significant for the cell wall fraction (*p* < 0.05); moreover, a statistical trend for the metabolite fraction was observed, suggesting that both the cell walls and metabolites contributed to the effects observed in the germinated spores (Figure 5D).

In contrast, the level of MCP-1 was reduced in inflamed cultures treated with the test products compared to LPS-treated control cultures (Figure 5B). The reduction in MCP-1 under inflamed culture conditions was highly significant for all three test products at the two middle doses (*p* < 0.01; Figure 5B).

### 3.4. Cytokines Involved in the Return to Homeostasis

**Figure 6:**
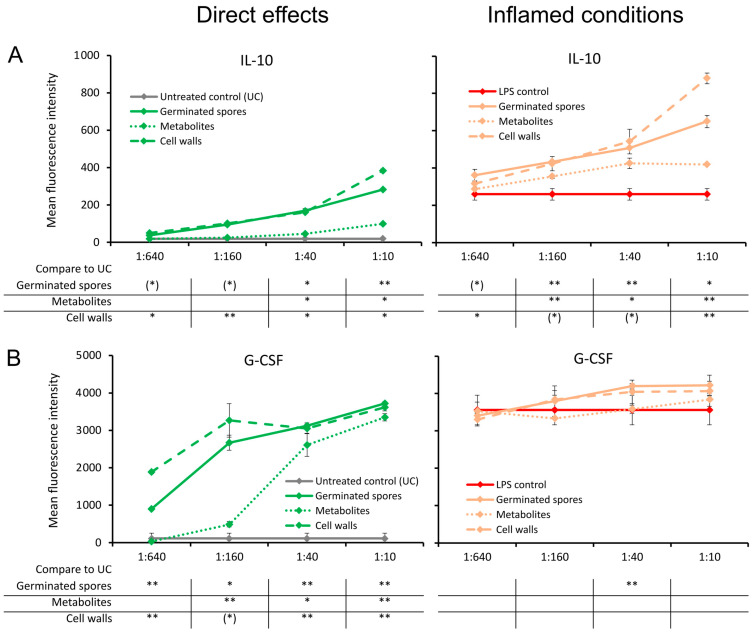
Cytokine production in cell cultures of human peripheral blood mononuclear cells. The left panel (green lines) shows the direct effects of the germinated spores, metabolites, and cell walls; untreated control cultures (UCs) were used as negative controls (grey lines). The right panel (orange lines) shows the effects of the test products under inflamed culture conditions, where inflammation was induced by lipopolysaccharides (LPSs); LPS alone was used as a positive control (red lines). (**A**) Interleukin-10 (IL-10) was induced by all 3 products; under inflamed culture conditions, a robust increase was observed in the LPS-induced levels of IL-10. (**B**) Granulocyte colony-stimulating factor (G-CSF) was robustly induced by all 3 products; under inflamed culture conditions, the germinated spores and the cell walls triggered an increase in the LPS-induced levels of G-CSF. Statistical comparisons are shown in the tables below each graph. For the graphs on the left side, the statistical comparisons are for the untreated control cultures (UCs). For the graphs on the right side, the statistical comparisons are for the LPS-treated control cultures (LPS). The statistical significance when comparing the test products to controls is indicated with (*) for *p* < 0.1, * for *p* < 0.05, and ** for *p* < 0.01.

The germinated spores and the cell wall fraction, and to a lesser extent the metabolite fraction, triggered a dose-dependent upregulation of the anti-inflammatory cytokine IL-10 under normal culture conditions (Figure 6A). The increase was highly significant for the germinated spores at the highest dose (*p* < 0.01) and significant for the cell walls and metabolites (*p* < 0.05) when compared to the untreated control cultures (Figure 6A). Under inflamed culture conditions, an increase in the LPS-induced production of IL-10 was observed, reaching a high level of statistical significance for the second-highest dose of germinated spores and for the highest dose of the cell walls and metabolites (*p* < 0.01; Figure 6A).

Compared to the untreated control cultures, the germinated spores and the cell wall fraction, and to a lesser extent the metabolite fraction, also triggered a dose-dependent upregulation of the growth factor G-CSF, which is known to affect restorative functions via the effects on stem cell migration (Figure 6B). This was also observed under inflamed conditions of the germinated spores and the cell wall fraction. The increase in G-CSF levels was highly significant for the second dose of germinated spores (*p* < 0.01) compared to the LPS control. In contrast, the metabolite fraction did not affect G-CSF levels under inflamed culture conditions (Figure 6B).

## 4. Discussion

Gut health has a definitive effect on physical and mental wellbeing. It requires a beneficial microbiome along the gut mucosal surfaces, where immune cells residing in the gut epithelium interact with the cell walls of commensal probiotic organisms and where secreted bacterial metabolites affect many aspects of health in the gut lumen and the underlying mucosal tissue. In order to maintain host health, gut bacteria and the human gut epithelial tissue must communicate. The immune cells inhabiting the gut mucosal tissue are an integral part of this crosstalk, and the postbiotic secreted metabolites are essential in this process [49].

This study examined the effects of the germinated spores of the novel probiotic strain *Bacillus coagulans* JBI-YZ6.3, alongside the metabolite and cell wall fractions, on immune activation and modulation in an unstressed versus inflamed environment. The goal was to test whether the immunological consequences of exposure to this probiotic bacterial strain would differ depending on the absence versus presence of inflammation. 

The results reported here have shown that in the absence of inflammation, both the cell wall and metabolite fractions from *B. coagulans* JBI-YZ6.3 triggered direct immune-activating effects. This was observed due to the up-regulation of both CD25 and CD69 activation markers and via the up-regulation of multiple immune-activating pro-inflammatory cytokines, including IL-1β, IL-6, IL-17, and TNF-α. For the cell wall fraction, immune-activating effects were expected based on the known engagement of cell wall peptidoglycans with Toll-Like receptor-2, which triggers the activation of immune cells via the NF-kβ and MAPK signaling pathways. The direct immune-activating properties of the metabolite fraction secreted by the germinated spores are more complex. The effects are likely mediated by multiple, distinct, non-overlapping mechanisms, some involving the modulation of inflammatory cell signaling pathways and others involving gene regulation via the induction of antioxidant response elements [46,50,51]. Consequently, the immune-activating effects caused by the secreted metabolites highlight the observation that various components of the metabolite fraction act synergistically to activate the immune system [47,48,52]. 

The changes to the expression of the immune cell activation markers CD25 and CD69 showed complexity and pointed to the intricate regulation of immune activity by *B. coagulans* JBI-YZ6.3. On NK cells, the expression levels of both CD25 and CD69 were increased significantly. The target cells that are recognized and killed by NK cells are not limited to cancer cells but also include virus-infected cells; thus, the effects of *B. coagulans* JBI-YZ6.3 on the NK cell activation status are clinically important. CD69 is rapidly induced in NK cells shortly after activation [53], and its direct role in NK cytotoxic activity has been demonstrated [54]. When human NK cells are co-cultured with K562 target cells, CD69 expression is upregulated, and the increase significantly correlated with NK cell activity, as measured by today’s gold standard CD107 mobilization assay [55]. CD69 has the capacity to activate the NK cytolytic machinery in the absence of other NK–target cell adhesion molecule interactions. Most importantly, a direct and highly significant correlation between CD69 levels and NK cell activity was demonstrated by Clausen’s team in a study involving 14 breast cancer patients tested repeatedly during chemotherapy [56]. 

On monocytes, CD25 expression levels were upregulated under normal culture conditions but downregulated under inflamed conditions, where the reduction was highly significant for germinated spores. Antigen-presenting cells of myeloid origin express CD25, the alpha chain of the IL-2 receptor, but they do not express the beta chain and hence are unresponsive to IL-2. However, they may compete for IL-2 and regulate T cell proliferation under inflamed conditions [57]. In contrast, CD69 was upregulated on monocytes both under normal and inflamed conditions. CD69 expression on monocytes has been functionally linked to the 5-lipoxygenase pathway, in which leukotrienes are produced [58]. Leukotrienes are important chemoattraction mediators, they are responsible for the rapid recruitment of immune cells relative to affected tissue [59], and the expression of CD69 on monocytes by *B. coagulans* JBI-YZ6.3 suggests that, in tissues, this would be associated with the recruitment of additional immune cells to the area.

Under inflamed culture conditions, the metabolite fraction showed immunomodulatory effects by stimulating the production of anti-inflammatory cytokines, such as IL-10, while also triggering a decrease in the pro-inflammatory chemokine MCP-1, suggesting that under inflamed conditions, there may be a reduced migration of inflammatory cells to tissue exposed to the bacterium and its metabolites. The metabolite fraction may offer a novel treatment strategy for immune support in patients with gut dysbiosis and a hostile gut microbiome. In some patients, consuming probiotics to change the composition of the gut microbiome may take time or be unsuccessful. Consuming the metabolite fraction, which is manufactured on a commercial production scale, may provide immediate health benefits independent of changes to the gut microbiome.

In previous studies on another strain of *B. coagulans* GBI-30 6086, the cell wall and secreted metabolite fractions demonstrated immunomodulatory effects by increasing the production of anti-inflammatory cytokines [43]. In another study, Benson et al. (2012) showed that the secreted metabolite fraction in the same strain supported the maturation of mononuclear phagocytes towards both macrophages and dendritic cells [44], highlighting their importance in protecting against foreign antigens.

Additionally, the upregulation of the stem cell-mobilizing growth factor G-CSF by germinated spores and cell wall components points to an effect on reparative functions. This is further supported by Jensen et al. (2017) [45], which showed that inactivated *B. coagulans* GBI-30 6086 induced a selective upregulation of G-CSF, which is known to be involved in post-injury and post-inflammation repair and regeneration. This suggests a beneficial role for *B. coagulans* JBI-YZ6.3 in the restoration of gut health and is the topic for ongoing studies in our lab.

From a methods perspective, we are presenting the details for isolating and testing the cell wall fraction and the postbiotic secreted metabolite fraction in parallel. This is due to an urgent need for standardized protocols and descriptive naming on this research topic [60]. One team compared the effects of the secreted metabolites to the bacterial lysate from *B. coagulans* and found significant differences between the two fractions on bone mass in ovariectomized rats [61]. Another team characterized the intracellular content of the *B. coagulans* GBI-30 strain, i.e., the bacterial lysate from which the cell walls were removed, but they did not compare the intracellular content to secreted metabolites [62]. While there is likely overlap between what the bacterium contains and what it is secreting, the two fractions are not identical, and we suggest that the intracellular content should not be defined as a postbiotic.

Notably, the results presented here demonstrated the superior immune-activating properties of the metabolically active, intact germinated spores when compared to the isolated cell wall and metabolite fractions. This was specifically observed for the upregulation of IL-1β and TNF-α under inflamed conditions and is likely due to synergistic actions of cell wall and metabolite components, which are present in cultures of the germinated spores. This work presents one method for testing the metabolically active bacterium in co-cultures with various cell types from the host.

The work presented here serves as a proof-of-concept study. Work is ongoing to evaluate the effects of cell walls and secreted metabolites on gut epithelial cells. It is of utmost importance to expand on the metabolomics analysis of secreted metabolites in order to better understand the potential for the support of gut health by this fraction. Further work should also characterize the immune-modulating effects on isolated immune cell types, including monocytes, NK cells, and T cells. It is also of interest to evaluate the effects on monocyte/macrophage polarization and dendritic cell maturation. Clinical studies on acute effects associated with consuming the metabolite fraction are also warranted.

## 5. Conclusions

The metabolically active probiotic bacterium *Bacillus coagulans* JBI-YZ6.31 showed direct immune-activating properties, and under inflamed conditions, it showed anti-inflammatory properties. Both the secreted metabolites and cell walls contributed to the immune-activating and anti-inflammatory properties of the novel probiotic strain *B. coagulans* JBI-YZ6.31. The cell wall fraction enhanced immune cell activation under inflamed conditions, while at the same time, it reduced the production of inflammatory cytokine TNF-α and increased the production of anti-inflammatory cytokine IL-10. This is of clinical importance, with the health-protective benefit of activating the immune defense along mucosal linings while at the same time modulating inflammatory reactions in a milieu widely exposed to potential pathogens.

## Data Availability

The data presented in this study are available upon reasonable request from the corresponding author.
